# Associations Between Musculoskeletal Structural Parameters and Pulmonary Function in Chronic Obstructive Pulmonary Disease: A Cross-Sectional Study

**DOI:** 10.3390/medsci14040476

**Published:** 2026-08-13

**Authors:** Kanogwun Thongchote, Panda Chainiramol, Pornpimol Muanjai, Sarawut Lapmanee

**Affiliations:** 1 Department of Physical Therapy, Faculty of Physical Therapy, Srinakharinwirot University, Nakorn Nayok 26120, Thailand; kanogwun@g.swu.ac.th (K.T.); tongpanda09@gmail.com (P.C.); 2 Department of Physical Therapy, Allied Health Sciences Faculty, Burapha University, Chonburi 20130, Thailand; pornpimolm@buu.ac.th; 3 Chulabhorn International College of Medicine, Thammasat University, Pathum Thani 12120, Thailand

**Keywords:** forward shoulder posture, lung disease, pulmonary rehabilitation, scapular distance, spirometry

## Abstract

**Background/Objectives**: This study aimed to compare structural and functional parameters between chronic obstructive pulmonary disease (COPD) patients and healthy controls, assess associations with pulmonary function, and identify factors independently associated with COPD status. **Methods**: A cross-sectional study was conducted in 89 participants (COPD: *n* = 45; controls: *n* = 44). Structural parameters included forward shoulder posture (FSP), scapular rotation, scapular distance (SD), and pectoralis minor index, measured bilaterally. Functional parameters included maximum inspiratory and expiratory pressures (MIP/MEP), chest expansion at three thoracic levels, spinal angles, and spirometry (%FVC, %FEV1, FEV1/FVC, FEF_25–75%_, PEFR). Univariate and stepwise multivariate logistic regression identified variables independently associated with COPD. **Results**: COPD patients were older and exhibited lower anthropometric measures. Scapular rotation was greater in COPD (*p* < 0.05 bilaterally), while SD was significantly reduced (*p* < 0.001 bilaterally). MIP, MEP, and chest expansion at all three levels were significantly impaired in COPD (*p* < 0.05). All spirometric parameters were markedly lower in COPD (all *p* < 0.001). SD was positively associated with spirometric indices on both sides (*p* < 0.01). FSP was inversely associated with MIP, MEP, and chest expansion (*p* < 0.05). In multivariate logistic regression, age (OR 1.280, *p* = 0.018), %FVC (OR 0.875, *p* = 0.029), and FEF_25–75%_ (OR 0.909, *p* < 0.001) were independently associated with COPD status. **Conclusions**: Patients with COPD demonstrated reduced SD and increased scapular rotation. SD was significantly associated with pulmonary function, whereas FEF_25–75%_ and %FVC emerged as independently associated variables of COPD status. Collectively, these findings underscore the clinical relevance of integrating targeted scapulothoracic and respiratory rehabilitation strategies to optimize functional outcomes and support overall respiratory health and well-being.

## 1. Introduction

Chronic obstructive pulmonary disease (COPD) is a heterogeneous lung condition characterized by chronic respiratory symptoms and persistent, often progressive, airflow obstruction due to abnormalities of the airways and/or alveoli [1]. It represents one of the leading causes of morbidity and mortality worldwide, accounting for approximately 3 million deaths annually and imposing enormous economic and societal burden [2]. Despite its classification as a respiratory disease, it is now firmly established that COPD exerts widespread systemic effects, including skeletal muscle dysfunction, nutritional abnormalities, and cardiovascular comorbidity [3].

Among the extrapulmonary manifestations of COPD, musculoskeletal changes in the thorax and shoulder girdle are increasingly recognized as contributors to functional decline. Lung hyperinflation, a hallmark of COPD, elevates the thoracic cage, alters the resting position of the scapulae, and modifies the length-tension relationships of the respiratory muscles and pectoral girdle [4,5]. These postural adaptations can, in turn, restrict thoracic mobility, impair the mechanical efficiency of the diaphragm and accessory inspiratory muscles, and perpetuate the cycle of dyspnea and physical deconditioning [6]. Improvements in chest wall posture and mobility are therefore incorporated into pulmonary rehabilitation guidelines [7].

Despite this rationale, quantitative characterization of specific musculoskeletal structural parameters, including scapular distance, scapular rotation, forward shoulder posture, spinal angle, and pectoralis minor index, and associations with objective spirometric parameters in COPD patients remains limited [5,6,7,8,9]. Although these morphological measures demonstrate only moderate reliability compared with advanced imaging techniques, such assessments are widely used in musculoskeletal and rehabilitation research because of low cost, non-invasive application, clinical feasibility, and the ability to provide clinically meaningful information on postural alterations associated with respiratory dysfunction [10,11,12]. Prior studies examining the upper quadrant in COPD have generally been constrained by small sample sizes, a limited range of structural measures, or the absence of systematic regression-based analyses linking musculoskeletal variables to individual lung function indices.

Importantly, alterations in lung volumes (e.g., reduced vital capacity and air trapping) and increased intrathoracic pressures associated with airflow limitation may contribute to adaptive changes in chest wall mechanics and shoulder girdle alignment. Under these conditions, scapular distance, a marker of scapular protraction reflecting anterior chest wall tightness and altered thoracic compliance, could be mechanistically linked to impaired respiratory mechanics. However, its relationship with a comprehensive range of spirometric indices has not been systematically evaluated in a well-characterized COPD cohort.

Furthermore, although FEF_25–75%_ is recognized as a sensitive marker of small airway obstruction and has been identified as an independent predictor of incident COPD development [13,14], its relationship to musculoskeletal parameters and its role as a predictor of COPD status in a clinical cohort including spirometric and demographic covariates has not been systematically evaluated.

The aims of this study were therefore (*i*) to compare a comprehensive array of musculoskeletal structural and functional parameters between clinically confirmed COPD patients and healthy controls; (*ii*) to examine the linear associations between structural variables of the shoulder girdle and thoracic region and objective pulmonary function parameters; and (*iii*) to identify structural parameters independently associated with COPD status using univariate and stepwise multivariate logistic regression. We hypothesized that individuals with COPD would demonstrate significant musculoskeletal alterations relative to healthy controls, that these structural parameters would be significantly associated with pulmonary function, and that a subset of these variables would independently characterize musculoskeletal alterations associated with COPD. Collectively, these findings are expected to provide clinically relevant insights to inform evidence-based decision-making and guide targeted intervention strategies aimed at optimizing cardiopulmonary health outcomes and reducing disease severity.

## 2. Materials and Methods

The study was conducted and reported in compliance with the STROBE (Strengthening the Reporting of Observational Studies in Epidemiology) guidelines for cross-sectional research. The study was conducted in accordance with the Declaration of Helsinki, and approved by the Institutional Review Board of Nakhon Nayok Hospital (REC 10/2560, date of approval: 25 January 2018) and the Nakhon Nayok Public Health Office (NPHO 2018-004, date of approval: 16 March 2018).

### 2.1. Participants

Participants were recruited from Nakhon Nayok Hospital and Banna Hospital in Nakhon Nayok province, Thailand. Inclusion criteria for the COPD group were (*i*) confirmed diagnosis of COPD per the Global Initiative for Chronic Obstructive Lung Disease (GOLD) 2023 criteria (post-bronchodilator FEV_1_/FVC < 0.70) [1]; (*ii*) clinically stable condition for at least four weeks prior to enrolment (i.e., no acute exacerbation); (*iii*) age ≥ 40 years; (*iv*) ability to provide informed consent and follow test instructions. Exclusion criteria included: recent thoracic or abdominal surgery (within one year); acute exacerbation of COPD at time of enrolment; neuromuscular or neurological conditions affecting thoracic mobility; severe cardiovascular disease contraindicating physical assessment; cognitive impairment; and any musculoskeletal condition of the upper limb or shoulder predating COPD diagnosis. Healthy controls were recruited from the same community and were age- and sex-matched to the COPD group to the extent possible. Controls were excluded if they had any respiratory disease, smoking history exceeding 10 pack-years, or significant musculoskeletal comorbidity. To account for the age imbalance between groups, we performed additional multivariable linear regression analyses with group as the independent variable and age included as a continuous covariate.

### 2.2. Structural Parameter Assessment

All measurements were performed by a trained assessor blinded to group allocation. Prior to participant recruitment, the examiner completed training and pilot measurements to standardize the assessment procedures. Previous studies have demonstrated acceptable intra-rater reliability for these measurements. The measurement procedures used in the present study followed previously validated protocols. Morphological measures such as forward shoulder posture, scapular posture, spinal angle, and pectoralis minor length have demonstrated excellent intra-rater reliability (ICC = 0.913–0.995). Anthropometric measurements were performed with participants in a relaxed sitting position. To ensure participant comfort, safety, and standardized measurement conditions, the same sitting protocol was applied to both the COPD and healthy control groups [15].

Bilateral assessments were conducted for all shoulder girdle parameters. Forward shoulder posture (FSP, cm) was assessed using the vernier height gauge (Mitutoyo 506-207, Mitutoyo Corporation, Kawasaki, Japan). Participants were measured as the horizontal distance from the posterior aspect of the acromion process to the assessment surface in a sitting position [16]. Scapular posture was assessed using the vernier caliper in sitting position [17]. Scapular rotation (SR, degrees) was defined as the angle of the inferior angle of the scapula relative to a vertical reference line. Scapular tilt (ST, degrees) was assessed as the anterior–posterior tilt angle of the scapular plane. Scapular distance was intended to quantify the resting scapulothoracic configuration under standardized conditions and was not designed to assess dynamic scapular protraction or scapulothoracic motion. Scapular distance (SD, cm) was measured as the perpendicular distance from the medial border of the scapula to the nearest thoracic spinous process at the level of the seventh thoracic vertebra. Pectoralis minor index (PMI) was calculated as the pectoralis minor muscle length divided by the subject’s height, multiplied by 100, as previously described [18]. The measurement was performed three times and calculated as the mean. 

### 2.3. Functional Parameter Assessment

Spinal angles at the cervical (C. angle), thoracic (T. angle), and lumbar (L. angle) levels were measured using a digital inclinometer [19]. Chest expansion (CE) was measured using a non-elastic tape at three levels, upper (axillary level), middle (xiphoid level), and lower (tenth rib level), as the difference in thoracic circumference between full inspiration and full expiration [20]. Respiratory muscle strength was assessed as maximum inspiratory pressure (MIP) and maximum expiratory pressure (MEP) using a respiratory pressure meter (Micro RPM, Micro Medical Ltd., Rochester, UK), following standardized American Thoracic Society/European Respiratory Society (ATS/ERS) procedures [21].

Spirometry was performed with a calibrated spirometer (Viasys Micro Lab 3500, Micro Medical Ltd., Rocheste, UK) following ATS/ERS 2019 technical standards [21]. Forced vital capacity (%FVC), forced expiratory volume in one second (%FEV_1_), FEV_1_/FVC ratio, forced expiratory flow at 25–75% of FVC (FEF_25–75%_), and peak expiratory flow rate (PEFR, L/min) were expressed as percentage predicted using reference equations appropriate for the study population [22].

### 2.4. Statistical Analysis

All statistical analyses were performed using R software version 4.2.0. The required sample size was determined a priori based on previously reported effect sizes for scapular parameters in patients with respiratory disease, with a target statistical power of 80% and a significance level of α = 0.05 [5]. Data distribution was evaluated using the Shapiro–Wilk test. Continuous variables with normal distributions were analyzed using the independent-samples *t*-test, whereas non-normally distributed variables were compared using the Wilcoxon rank-sum test. Data are presented as mean ± standard deviation (SD) or median [interquartile range (IQR)], according to data distribution. Associations between structural and functional variables were examined using Pearson’s or Spearman’s correlation coefficients, as appropriate. Linear regression models were constructed to evaluate the relationships between musculoskeletal structural parameters (independent variables) and respiratory functional outcomes (dependent variables). The assumptions of linear regression, including linearity, homoscedasticity, and normal distribution of residuals, were assessed prior to model interpretation. For identification of potential predictors, univariate logistic regression analyses were initially performed. Variables with a threshold of *p* < 0.10 in univariate analyses were entered into multivariable logistic regression models using backward elimination based on the Akaike Information Criterion (AIC). The final model performance was assessed using Nagelkerke’s R^2^ and the Hosmer–Lemeshow goodness-of-fit test. Because of the significant age difference between the COPD and healthy control groups, additional multivariable linear regression analyses were performed for each continuous outcome measure. Group status was entered as the primary independent variable, with age included as a continuous covariate to adjust for potential confounding effects. Adjusted between-group differences were reported as regression coefficients or adjusted mean differences with corresponding 95% confidence intervals (CIs). The group-by-age interaction term was examined to evaluate the assumption of homogeneity of regression slopes. All statistical tests were two-sided, and a *p*-value < 0.05 was considered statistically significant. Missing data were not imputed and were analyzed using available-case analysis.

## 3. Results

### 3.1. Participant Characteristics and Between-Group Comparisons

Complete data were available for 89 participants: 45 patients with confirmed COPD and 44 healthy controls. Baseline physical and clinical characteristics are presented in Table 1. COPD patients were significantly older (72.8 ± 8.1 vs. 66.0 ± 5.2 years; *p* < 0.001), lighter, shorter, and had significantly lower BMI compared to controls.

Regarding structural variables, FSP did not differ significantly between groups on either side (*p* > 0.05). In contrast, scapular upward rotation was significantly greater in the COPD group bilaterally (left: 6.64 ± 2.8 vs. 5.42 ± 2.1, *p* = 0.022; right: 6.52 ± 2.6 vs. 5.27 ± 2.0, *p* = 0.006). ST showed no significant between-group differences (*p* > 0.05 bilaterally). SD was significantly reduced in COPD patients on both sides (left: 6.42 ± 0.50 vs. 7.09 ± 0.84 cm, *p* = 0.001; right: 6.54 ± 0.56 vs. 7.18 ± 0.83 cm, *p* < 0.001). Additionally, the PMI was significantly lower on the right side in the COPD group (*p* = 0.041), with no significant difference observed on the left.

Respiratory muscle strength was significantly impaired in COPD patients, as evidenced by lower MIP (62.1 ± 27 vs. 84.0 ± 28 cmH_2_O, *p* < 0.001) and MEP (96.1 ± 34 vs. 114 ± 34 cmH_2_O, *p* = 0.018). Chest wall expansion was also significantly reduced at all assessed levels (*p* < 0.05). However, no significant differences were observed in spinal curvature angles at the cervical, thoracic, or lumbar regions (*p* > 0.05) (Table 1).

All spirometric indices were significantly diminished in the COPD group, including FEV_1_/FVC ratio, %FEV_1_, %FVC, FEF_25–75%_, and peak expiratory flow rate (PEFR) (all *p* < 0.001) (Table 1).

### 3.2. Age-Adjusted Analysis

Because the COPD group was significantly older than the healthy control group (72.8 ± 8.1 vs. 66 ± 5.2 years), multivariable linear regression analyses were performed for each continuous outcome, with age included as a covariate to determine whether group differences persisted independent of age.

After adjustment for age, spirometric parameters remained markedly and significantly lower in the COPD group compared with controls, including FVC% (*β* = −26.7, 95% CI −35.2 to −18.2, *p* < 0.001), FVC volume (*β* = −0.92 L, *p* < 0.001), FEV_1_ (*β* = −54.2, *p* < 0.001), FEV_1_/FVC ratio (*β* = −31.8, *p* < 0.001), FEF_25–75%_ (*β* = −87.7, *p* < 0.001), PEFR (*β* = −43.0, *p* < 0.001), and Vext (*β* = −1.71, *p* < 0.001). These findings indicate that the observed pulmonary function deficits in COPD patients are not attributable to the age difference between groups.

Chest wall and thoracic configuration measures similarly showed group differences that persisted after age adjustment. Sternal depression was significantly reduced on both sides (SD_Lt: *β* = −0.70, *p* < 0.001; SD_Rt: *β* = −0.60, *p* = 0.001), as was the pectoralis minor index (PMI_Lt: *β* = −0.48, *p* = 0.042; PMI_Rt: *β* = −0.73, *p* = 0.009). Chest expansion measures were also significantly lower across all three levels (CE_upper: *β* = −1.00, *p* < 0.001; CE_Middle: *β* = −1.07, *p* = 0.004; CE_Lower: *β* = −1.77, *p* < 0.001). MIP remained significantly reduced in COPD after adjustment (*β* = −15.0, *p* = 0.024); notably, age itself was also an independent significant contributor to MIP variability (*β* = −1.54, *p* = 0.001), indicating that lower inspiratory muscle strength reflects both disease status and the aging process.

In contrast, several measures that appeared different on unadjusted comparison were attenuated after controlling for age and did not reach conventional significance, including FSP_Lt/Rt, ST_Lt/Rt, MEP, and the C, T, and L spinal angles (all *p* > 0.05), while SR_Rt remained borderline significant (*β* = 1.33, *p* = 0.029) and SR_Lt approached but did not reach significance (*p* = 0.084). This suggests that age may be a partial confounder for these particular chest-wall and postural parameters, and that the crude between-group differences observed for these variables should be interpreted with caution.

Taken together, these age-adjusted analyses indicate that the major pulmonary function deficits and several chest-wall deformity indices remained significantly associated with COPD after adjustment for age, whereas age-related confounding remained evident for several postural variables.

### 3.3. Associations Between Structural and Functional Parameters

Significant linear associations between structural and functional parameters are summarized in Table 2. FSP demonstrated consistent inverse relationships with indices of respiratory muscle strength and chest wall mobility. Specifically, greater FSP on either side was associated with lower MIP (left: *β* = −0.017, *p* = 0.007; right: *β* = −0.015, *p* = 0.011), reduced MEP (left: *β* = −0.011, *p* = 0.041), and diminished chest expansion across all three anatomical levels (all *p* < 0.05).

Among the structural variables, SD exhibited the most robust and consistent associations with pulmonary function. Greater SD bilaterally was significantly and positively associated with %FVC, %FEV_1_, FEF_25–75%_, and peak expiratory flow rate (PEFR) (all *p* < 0.01), as well as with the FEV_1_/FVC ratio (left: *β* = 0.013, *p* = 0.002; right: *β* = 0.011, *p* = 0.014). Notably, both left and right SD were significantly associated with key spirometric indices, particularly %FVC and FEF_25–75%_, which are themselves strongly linked to COPD status.

Furthermore, right-sided scapular rotation demonstrated a modest but significant inverse association with the FEV_1_/FVC ratio (*p* = 0.040), as detailed in Table 2. Meanwhile, PMI was also inversely associated with cervical angle (left: *β* = −0.038, *p* = 0.014; right: *β* = −0.036, *p* = 0.045) and PMI Right showed a positive association with thoracic angle (*β* = 0.023, *p* = 0.044), suggesting a link between pectoral tightness and cervical–thoracic postural configuration.

### 3.4. Variables Associated with COPD Status

Univariate and multivariate logistic regression results are presented in Table 3. In the univariate models, age, height, weight, BMI, MIP, MEP, chest expansion at all three anatomical levels, %FVC, %FEV_1_, FEV_1_/FVC ratio, FEF_25–75%_, FEF_25–75%_ volume, PEFR, PEFR volume, and extrapolated volume were all identified as significant variables associated with COPD status (all *p* < 0.05). Subsequent stepwise multivariate logistic regression with AIC-based model selection retained three variables as independently associated with COPD status: age, %FVC, and FEF_25–75%_ (Table 3).

**Figure 1:**
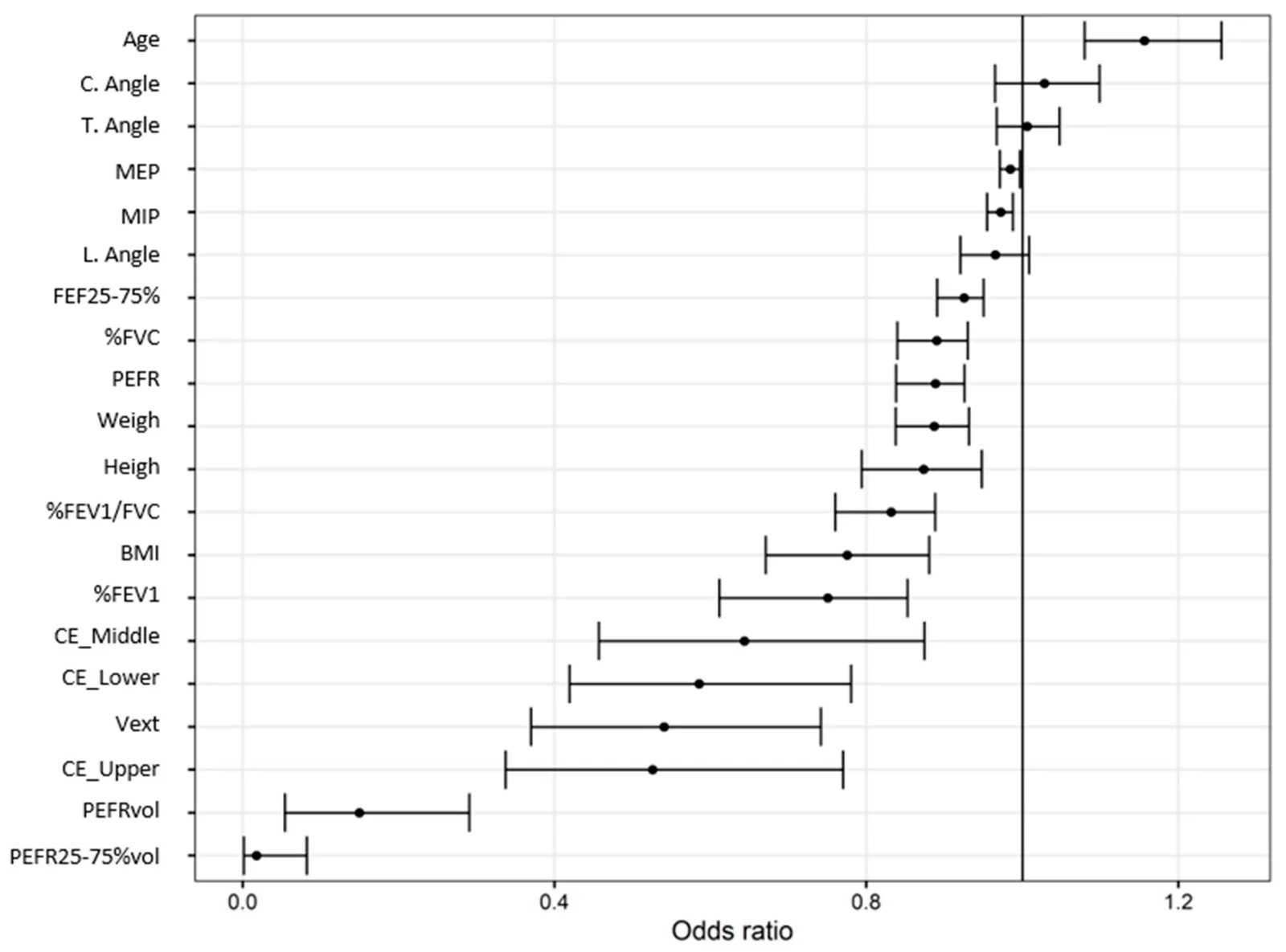
Variables independently associated with COPD from univariate logistic regression analysis. The odds ratios (ORs) with CIs derived from univariate logistic regression analyses of variables associated with COPD status. Each point estimate represents the OR, while the horizontal bars denote the 95% CI.

The forest plot illustrating univariate odds ratios is presented in Figure 1. Notably, FEF_25–75%_ demonstrates an OR below 1 with a 95% CI that does not cross unity, indicating a statistically significant inverse association with COPD. This finding suggests that greater small airway function, as reflected by higher FEF_25–75%_ values, is associated with reduced odds of COPD, supporting its utility as a discriminative functional parameter in this population.

## 4. Discussion

This cross-sectional study comprehensively characterized musculoskeletal structural parameters, respiratory muscle strength, chest wall expansion, and pulmonary function in a clinically confirmed COPD cohort compared to healthy controls. The principal novel findings were (*i*) bilateral SD was significantly reduced in COPD patients, and was positively and consistently associated with %FVC, %FEV_1_, FEF_25–75%_, and PEFR; (*ii*) FSP was inversely associated with respiratory muscle strength and chest expansion, independent of group; (*iii*) FEF_25–75%_ and %FVC emerged as independent associated variables of COPD status in multivariate analysis alongside age. These findings advance understanding of the structural–functional relationships in COPD and have implications for physiotherapy assessment and rehabilitation planning.

Reduced SD in COPD represents a mechanistically plausible finding within the context of disease pathophysiology. Lung hyperinflation, a hallmark consequence of expiratory airflow limitation, promotes a barrel-shaped thoracic configuration, elevates the rib cage, and alters resting scapular alignment through changes in scapulothoracic contact mechanics. Consistent with prior meta-analytic evidence, patients with COPD commonly exhibit scapular elevation, internal rotation, protraction, and anterior tilt in static postures [22]. A reduction in scapular distance observed in the present study reflects the resting anatomical relationship between the scapula and thoracic spine under standardized measurement conditions. Because this measure is influenced by thoracic cage morphology, resting scapular orientation, soft tissue characteristics, and age-related postural adaptations, it should not be interpreted as representing scapular protraction or medial displacement alone. Such alterations may compromise the mechanical advantage and force-generating capacity of periscapular muscles involved in accessory respiratory function, including the serratus anterior and rhomboids. Supporting this interpretation, previous interventional studies have demonstrated that scapulothoracic-focused exercise programs, incorporating pectoralis muscle stretching alongside strengthening of the serratus anterior and lower trapezius, can improve respiratory muscle strength and chest wall mobility in COPD patients presenting with FSP [6]. Furthermore, phenotypic variability within COPD should be considered. Individuals with the emphysematous phenotype tend to exhibit more pronounced postural malalignments, including forward head and shoulder posture, increased thoracic kyphosis, scapular elevation, and asymmetry, compared with those with chronic bronchitis and age-matched healthy controls [23]. These distinctions underscore the importance of incorporating phenotype-specific considerations into comprehensive assessment and the design of targeted, individualized intervention strategies in COPD management.

The consistent positive associations between SD and FEF_25–75%_ are particularly noteworthy. FEF_25–75%_, representing forced mid-expiratory flow, is widely regarded as a sensitive—albeit variable—indicator of small airway function [24]. Kwon et al. [13] also demonstrated that low FEF_25–75%_ in individuals with normal spirometry predicted the development of COPD over a 10-year follow-up, with a hazard ratio of 3.31. A multinational analysis further confirmed that isolated small airways obstruction, detectable by FEF_25–75%_, significantly increased the odds of future chronic airflow obstruction [13]. In the present study, FEF_25–75%_ emerged as the strongest independent predictor of COPD status in multivariate analysis (OR = 0.909 per unit increase, *p* < 0.001), reinforcing its discriminative utility within a cross-sectional clinical cohort. The observed structural–functional relationship between SD and FEF_25–75%_ suggests a plausible biomechanical pathway, whereby reduced scapulothoracic spacing may exacerbate small airway flow limitation through constraints on dynamic thoracic expansion during expiration.

FSP demonstrated significant inverse associations with MIP, MEP, and chest expansion across all three thoracic levels. These findings are consistent with established biomechanical principles, whereby scapular protraction and anterior tilt disrupt the optimal length–tension relationship of the pectoralis minor and impair coordinated activation of scapular stabilizers, thereby limiting rib cage excursion and ventilatory mechanics [25].

Consistent with these observations, healthy controls exhibited significantly greater scapular distance, pectoralis minor index, chest wall mobility, and respiratory muscle strength than participants with COPD. These differences likely reflect the chronic postural and respiratory adaptations associated with COPD. Persistent lung hyperinflation elevates the resting position of the rib cage and thoracic spine, promoting thoracic kyphosis, forward shoulder posture, and scapular protraction, which contribute to adaptive shortening of the pectoralis minor and reduced scapular distance [26]. Chronic hyperinflation also increases chest wall stiffness and restricts thoracic expansion, resulting in reduced chest mobility. Consequently, the diaphragm and accessory inspiratory muscles operate at a shortened resting length, diminishing their mechanical advantage according to the length–tension relationship [27]. These biomechanical adaptations likely account for the significantly lower inspiratory and expiratory muscle strength observed in the COPD group.

Taken together, these findings suggest that altered scapular posture, pectoralis minor shortening, restricted chest wall mobility, and respiratory muscle weakness are interrelated manifestations of chronic thoracic hyperinflation in COPD. The structural adaptations of the thorax not only affect musculoskeletal alignment but also compromise respiratory mechanics, ultimately reducing respiratory muscle performance.

The observed reductions in chest expansion across all levels in COPD patients align with evidence from a recent randomized controlled trial by Tsui et al. [28], which reported a positive association between chest wall expansion and pulmonary function and demonstrated significant improvements following targeted chest wall mobilization in individuals with severe COPD. In addition, prior work has shown that scapulothoracic-focused exercise interventions—incorporating pectoralis stretching and strengthening of key stabilizers—can reduce FSP and enhance respiratory muscle performance. These effects are likely mediated through increased anterior chest wall compliance via lengthening of the pectoral and intercostal musculature, improved rib–pelvis alignment, reduced intra-abdominal pressure, and facilitation of caudal diaphragmatic excursion [6]. Despite these functional associations, FSP did not differ significantly between groups in the present study. This apparent discrepancy may reflect measurement ceiling effects using tape measures or heterogeneity in disease severity within the COPD cohort. An additional explanation may relate to phenotypic variation; specifically, the sample may have included a lower proportion of emphysematous phenotypes, which are typically characterized by more pronounced FSP due to greater reliance on accessory cervical musculature. In such cases, compensatory overactivation of the sternocleidomastoid, secondary to diaphragmatic dysfunction, may contribute to altered neck muscle efficiency and more evident postural deviations [23]. Although MIP and MEP remain standard measures of respiratory muscle strength, they do not characterize postural alignment or shoulder girdle mechanics. The additional musculoskeletal assessments evaluated in the present study may therefore provide complementary information regarding movement impairments that could be considered during individualized physical therapy assessment.

The absence of significant differences in spinal curvature between groups warrants consideration. While thoracic kyphosis has been reported in some COPD populations, it is not universal [5], and may be more pronounced in advanced or long-standing disease [23]. In the present sample, mean thoracic kyphosis angles in both groups fell within broadly normative ranges, suggesting that sagittal spinal alignment may be a less sensitive or discriminative structural marker compared with scapular parameters in this population. These findings imply that alterations in scapulothoracic mechanics, rather than global spinal alignment, may play a more prominent role in the musculoskeletal manifestations associated with COPD in relatively heterogeneous or less severe cohorts.

The identification of %FVC, FEF_25–75%_, and age as independently associated with COPD status is consistent with established epidemiological and physiological evidence. Age-related decline in pulmonary function reduces respiratory reserve, and advancing age interacts synergistically with obstructive pathology to exacerbate functional impairment [29]. Emerging evidence further indicates that ageing, frailty, and COPD do not occur in isolation but represent interrelated processes characterized by diminished immune competence, impaired pulmonary defense mechanisms, and progressive reductions in muscle mass and strength, including the development of sarcopenia and increased susceptibility to fatigue in advanced disease [30]. The independent contribution of %FVC should be interpreted with caution. While reductions in %FVC may reflect the volumetric constraint imposed by air trapping and lung hyperinflation—even in the presence of otherwise preserved spirometry—the most clinically valid indices of hyperinflation remain static lung volume measures, particularly the residual volume to total lung capacity ratio (RV/TLC) obtained via body plethysmography. Recent work has also demonstrated the utility of predictive nomograms incorporating %FEV_1_ and %FVC to estimate RV/TLC, providing a pragmatic surrogate for detecting air trapping in settings where direct measurement is not feasible [31]. In parallel, FEF_25–75%_ captures dysfunction within the peripheral airways, which may precede the onset of more overt airflow limitation detectable by conventional spirometric indices [14,32,33,34]. Notably, the absence of anthropometric and structural variables in the final multivariate model suggests that spirometric measures account for the majority of disease-related variance once demographic factors and structural chest configuration are considered. This finding underscores the dominant role of functional respiratory parameters in discriminating COPD status within this cohort.

Several limitations of this study should be acknowledged. First, the cross-sectional design precludes causal inference regarding the temporal relationship between musculoskeletal alterations and pulmonary dysfunction. Longitudinal studies are needed to determine whether these structural changes precede, accompany, or result from disease progression.

Second, the COPD group was older than the healthy control group and was not matched for age. Because aging independently affects posture, muscle strength, and thoracic mobility, age may have contributed to the observed between-group differences. Although the analyses were adjusted for age, residual confounding cannot be excluded. In addition, hand dominance was not recorded or matched between groups. As upper limb dominance may influence scapular posture, future studies should account for this potential confounder through matching or statistical adjustment.

Third, although the morphological assessments used in this study have lower reliability than radiographic or three-dimensional motion analysis techniques, they remain widely accepted in musculoskeletal and rehabilitation research because they are inexpensive, non-invasive, clinically feasible, and suitable for routine practice. Nevertheless, the findings should be interpreted with consideration of the inherent measurement error associated with these assessments.

Fourth, GOLD stage, smoking history, disease duration, nutritional status, medication use, and comorbidity burden were not included in the analyses. These factors may influence musculoskeletal adaptations and respiratory function and should be incorporated into future studies to improve clinical interpretation.

Finally, the moderate sample size and single-center recruitment from a limited geographical region may restrict the statistical power and generalizability of the findings. Larger multicenter, longitudinal studies with closely age-matched controls and COPD severity stratification are needed to validate these findings and determine how musculoskeletal alterations evolve across different stages of disease progression.

## 5. Conclusions

This study demonstrated that patients with COPD exhibited reduced scapular distance, increased scapular rotation bilaterally, impaired respiratory muscle strength, and reduced chest expansion compared with healthy controls. These findings suggest that chronic postural adaptations are closely associated with altered respiratory mechanics in COPD. Scapular distance was positively associated with several pulmonary function parameters, suggesting that it reflects musculoskeletal adaptations accompanying pulmonary impairment in COPD rather than serving as a clinical marker associated with COPD. In multivariable analysis, FEF_25–75%_ and %FVC remained independently associated with COPD status after adjustment for age, highlighting the clinical relevance of small airway function measures in the comprehensive assessment of individuals with COPD. These findings support the routine inclusion of comprehensive shoulder girdle, posture, chest wall mobility, and respiratory muscle function as part of physiotherapy evaluation in individuals with COPD. These musculoskeletal characteristics may complement clinical assessment and rehabilitation planning but should not be interpreted as diagnostic markers of COPD. The observed postural alterations highlight the potential importance of addressing shoulder girdle dysfunction during musculoskeletal rehabilitation, although the effectiveness of specific scapular-focused interventions requires further investigation. The rehabilitation programs may benefit from including the assessment and management of postural abnormalities and chest wall mobility alongside conventional interventions. Such an approach may contribute to optimizing breathing mechanics and overall physical function. From a clinical perspective, these findings suggest that assessment of shoulder girdle posture, chest wall mobility, and respiratory muscle function may provide complementary information during comprehensive physical therapy evaluation of individuals with COPD. The observed musculoskeletal alterations highlight potential targets for individualized rehabilitation alongside conventional pulmonary rehabilitation. However, the effectiveness of specific posture- or scapular-focused interventions should be confirmed in prospective interventional studies. Recognition of these impairments may assist physical therapists in tailoring rehabilitation strategies aimed at optimizing breathing mechanics and improving functional performance. Prospective studies with larger samples, GOLD staging, and longitudinal follow-up are needed to validate and extend these findings. Future research should determine whether musculoskeletal alterations vary with COPD severity, disease duration, nutritional status, and regional lung hyperinflation, and evaluate whether targeted rehabilitation strategies can improve respiratory mechanics and functional outcomes.

## Figures and Tables

**Table 1:** Physical, anthropometric, structural, and functional characteristics of COPD patients and healthy controls.

Category	Parameter	COPD (*n* = 45)	Controls (*n* = 44)	*p*-Value
General characteristics	Age (years)	72.8 ± 8.1	66.0 ± 5.2	<0.001 *
	Height (cm)	161 ± 6.7	165 ± 5.2	0.003 *
	Weight (kg)	55.6 ± 9.0	68.5 ± 11.2	<0.001 *
	BMI (kg/m^2^)	21.5 ± 3.5	24.7 ± 3.6	<0.001 *
Forward shoulder posture (cm)	Left	7.94 ± 1.8	8.09 ± 1.8	0.743
	Right	8.18 ± 1.9	8.13 ± 1.5	0.931
Scapular rotation (°)	Left	6.64 ± 2.8	5.42 ± 2.1	0.022 *
	Right	6.52 ± 2.6	5.27 ± 2.0	0.006 *
Scapular tilt (°)	Left	34.4 ± 5.9	36.1 ± 6.2	0.193
	Right	35.0 ± 6.0	35.8 ± 5.7	0.703
Scapular distance (cm)	Left	6.42 ± 0.50	7.09 ± 0.84	0.001 *
	Right	6.54 ± 0.56	7.18 ± 0.83	<0.001 *
Pectoralis minor index	Left	11.5 ± 0.93	11.7 ± 0.96	0.052
	Right	11.32 ± 0.89	11.8 ± 1.27	0.041 *
Respiratory muscle strength (cmH_2_O)	MIP	62.1 ± 27	84.0 ± 28	<0.001 *
	MEP	96.1 ± 34	114 ± 34	0.018 *
Chest expansion (cm)	Upper	3.97 ± 1.1	4.86 ± 1.3	<0.001 *
	Middle	3.71 ± 1.2	4.58 ± 1.6	0.010 *
	Lower	3.85 ± 1.4	5.20 ± 1.8	<0.001 *
Spinal angle (°)	Cervical	42.9 ± 6.7	41.7 ± 6.4	0.45
	Thoracic	36.3 ± 11	35.6 ± 9.9	0.806
	Lumbar	34.8 ± 9.7	37.9 ± 9.5	0.177
Lung function (% predicted)	%FVC	69.9 ± 18	97.1 ± 14	<0.001 *
	%FEV_1_	58.7 ± 21	109 ± 15	<0.001 *
	FEV_1_/FVC	78.7 ± 16	107 ± 7.7	<0.001 *
	FEF_25–75%_	36.0 ± 27	112 ± 29	<0.001 *
	PEFR (L/min)	50.2 ± 21	90.4 ± 14	<0.001 *

Data are mean ± SD unless otherwise stated. Boldface *p*-values indicate statistical significance (*p* < 0.05). BMI = body mass index; MIP = maximum inspiratory pressure; MEP = maximum expiratory pressure; FVC = forced vital capacity; FEV_1_ = forced expiratory volume in 1 s; FEF_25–75%_ = forced expiratory flow at 25–75% FVC; PEFR = peak expiratory flow rate. * *p* < 0.05.

**Table 2:** Summary of significant linear regression associations between structural parameters and functional parameters (*n* = 89).

Structural Parameters	Functional Parameters	Estimated Effect (95% CI)	Adjusted *p*-Value
FSP Left	MIP	−0.017 (−0.030, −0.005)	0.007
FSP Right	MIP	−0.015 (−0.027, −0.003)	0.011
FSP Left	MEP	−0.011 (−0.022, −0.001)	0.041
FSP Left	CE Upper	−0.416 (−0.708, −0.124)	0.006
FSP Right	CE Upper	−0.393 (−0.671, −0.115)	0.006
FSP Left	CE Middle	−0.411 (−0.658, −0.164)	0.001
FSP Right	CE Middle	−0.397 (−0.631, −0.162)	0.001
FSP Left	CE Lower	−0.220 (−0.438, −0.001)	0.049
SD Left	%FVC	0.015 (0.008, 0.022)	<0.001
SD Right	%FVC	0.014 (0.007, 0.021)	<0.001
SD Left	%FEV_1_	0.011 (0.006, 0.016)	<0.001
SD Right	%FEV_1_	0.009 (0.004, 0.014)	<0.001
SD Left	FEF_25–75%_	0.006 (0.003, 0.009)	<0.001
SD Right	FEF_25–75%_	0.005 (0.001, 0.008)	0.005
SD Left	PEFR	0.010 (0.004, 0.016)	<0.001
SD Right	PEFR	0.010 (0.005, 0.016)	<0.001
SR Right	FEV_1_/FVC	−0.028 (−0.055, 0.001)	0.040

Only associations reaching *p* < 0.05 are shown. CI = confidence interval; FSP = forward shoulder posture; SD = scapular distance; SR = scapular rotation; CE = chest expansion; MIP = maximum inspiratory pressure; MEP = maximum expiratory pressure; FVC = forced vital capacity; FEV_1_ = forced expiratory volume in 1 s; FEF_25–75%_ = forced expiratory flow at 25–75% FVC; PEFR = peak expiratory flow rate.

**Table 3:** Univariate and stepwise multivariate logistic regression analysis for variables independently associated with COPD.

Variable	Univariate OR (95% CI)	*p*-Value	Multivariate OR (95% CI)	*p*-Value
Age (years)	1.156 (1.080–1.255)	<0.001 *	1.280 (1.093–1.500)	0.018 *
Height (cm)	0.873 (0.794–0.948)	0.003 *	—	—
Weight (kg)	0.887 (0.837–0.931)	<0.001 *	—	—
BMI (kg/m^2^)	0.775 (0.671–0.880)	<0.001 *	—	—
MIP	0.972 (0.954–0.988)	0.001 *	—	—
MEP	0.984 (0.971–0.997)	0.017 *	—	—
CE Upper	0.526 (0.337–0.770)	0.002 *	—	—
CE Middle	0.643 (0.457–0.875)	0.007 *	—	—
CE Lower	0.585 (0.419–0.780)	0.001 *	—	—
C. angle	1.028 (0.965–1.099)	0.396	—	—
T. angle	1.006 (0.967–1.047)	0.768	—	—
L. angle	0.965 (0.920–1.009)	0.124	—	—
%FVC	0.890 (0.839–0.930)	<0.001 *	0.875 (0.750–0.962)	0.029 *
%FEV_1_	0.751 (0.611–0.853)	0.001 *	—	—
%FEV_1_/FVC	0.832 (0.760–0.888)	<0.001 *	—	—
FEF_25–75%_	0.925 (0.891–0.950)	<0.001 *	0.909 (0.841–0.949)	<0.001 *
PEFR	0.888 (0.838–0.926)	<0.001 *	—	—
Vext	0.541 (0.370–0.742)	<0.001 *	—	—

OR = odds ratio; CI = confidence interval; CE = chest expansion; MIP = maximum inspiratory pressure; MEP = maximum expiratory pressure; FVC = forced vital capacity; FEV_1_ = forced expiratory volume in 1 s; FEF_25–75%_ = forced expiratory flow at 25–75% FVC; PEFR = peak expiratory flow rate; Vext = extrapolated volume. * *p* < 0.05.

## Data Availability

The data presented in this study are available upon request from the corresponding author, consistent with institutional policies and applicable privacy regulations.

## Abbreviations

The following abbreviations are used in this manuscript:

COPD	Chronic Obstructive Pulmonary Disease
FSP	Forward Shoulder Posture
SR	Scapular Rotation
ST	Scapular Tilt
SD	Scapular Distance
PMI	Pectoralis Minor Index
MIP	Maximum Inspiratory Pressure
MEP	Maximum Expiratory Pressure
CE	Chest Expansion
C. angle	Cervical Angle
T. angle	Thoracic Angle
L. angle	Lumbar Angle
FVC	Forced Vital Capacity
%FVC	Percent Predicted Forced Vital Capacity
FEV_1_	Forced Expiratory Volume in One Second
%FEV_1_	Percent Predicted Forced Expiratory Volume in One Second
FEV_1_/FVC	Ratio of Forced Expiratory Volume in One Second to Forced Vital Capacity
FEF_25–75%_	Forced Expiratory Flow at 25–75% of Forced Vital Capacity
PEFR	Peak Expiratory Flow Rate
BMI	Body Mass Index
OR	Odds Ratio
CI	Confidence Interval
ATS	American Thoracic Society
ERS	European Respiratory Society
GOLD	Global Initiative for Chronic Obstructive Lung Disease
STROBE	Strengthening the Reporting of Observational Studies in Epidemiology
AIC	Akaike Information Criterion
REC	Research Ethics Committee
NPHO	Nakhon Nayok Public Health Office
RV/TLC	Residual Volume to Total Lung Capacity Ratio
SDG	Sustainable Development Goal
UK	United Kingdom
cmH_2_O	Centimeters of Water Pressure
